# Let’s (not) talk about synthetic biology: Framing an emerging technology in public and stakeholder dialogues

**DOI:** 10.1177/0963662520907255

**Published:** 2020-03-03

**Authors:** Anja Bauer, Alexander Bogner

**Affiliations:** University of Klagenfurt, Austria; Austrian Academy of Sciences, Austria; Austrian Academy of Sciences, Austria

**Keywords:** frames, framing, Responsible Research and Innovation, societal engagement, synthetic biology

## Abstract

Synthetic biology is an emerging technoscience, which, so far, lacks a broader public debate. To foster early societal dialogue, a range of public engagement events have been initiated over the past decade. This article discusses the configurations of the emerging debate on synthetic biology in the context of the EU FP7 project SYNENERGENE. Drawing on notions of frames and framing in media studies and policy analysis, we ask which distinct frames are invoked and become dominant in current discussions about synthetic biology. Our analysis indicates significant reconfigurations in the framing of synthetic biology compared with previous biotechnology debates. Frames that traditionally served to problematize biotechnology, that is, ethics, risks, and economics, become less dominant. Instead, the potential to contribute to social progress is placed in the foreground. Moreover, discussions on ethics, risks, and governance frequently occur on an abstract level, invoking generic statements that could be made for any new technology.

## 1. Introduction

As long as in the prosperous industrial society of the post-war period technical progress was equated with economic and social progress, there was no reason to problematize new technologies (Habermas, 1970). Technical development appeared as an autonomous process and technology itself as something monolithic, unambiguous, and predetermined. This conception of technological progress has changed significantly with rising awareness of the unintended and unforeseen side-effects, increasing risks, and fundamental ethical questions surrounding science, technology, and innovation. New technologies have increasingly become the subject of societal conflicts (Bogner and Torgersen, 2015; Torgersen and Schmidt, 2013), in which opposing visions, hopes, and fears collide. The governance concept of Responsible Research and Innovation (RRI) aims at aligning science and technology development with societal needs, values, and expectations. The call for societal engagement is at the core of the RRI concept: societal actors should be engaged early on in debates about research and technological developments (Owen et al., 2012; von Schomberg, 2013).

Which pictures of new technologies are (re)produced in such societal engagement processes? Technologies are open to a variety of competing interpretations and interests (Pinch and Bijker, 1984). There is no such thing as a technology’s ‘essence’ or identity; rather, what technologies mean is the subject of ongoing negotiations in which past technological controversies play just as much a role as visions of the future and socio-technical imaginaries (Jasanoff and Kim, 2015), governance regimes, and public awareness (Bijker, 2012). As a result, the public discourse gives technology a meaning that is neither random nor merely mirrors an issue that is given ex ante (Bogner and Torgersen, 2015: 519). Moreover, each meaning has different consequences regarding research and innovation, actor constellations, and governance.

In our contribution, we discuss the configurations of the emerging debate on synthetic biology (synbio) in the context of professionally organized participation events (‘invited participation’). Synbio is an emerging research and technology area focusing on the design and construction of new biological parts, devices, and systems and the redesign of existing natural biological systems for useful purposes (Endy, 2005). So far, this research area is little known and debated among wider publics. Hence, it is still unclear what public concerns, interests, and values are elicited by this emerging technology. To proactively address public concerns and potential conflicts, a range of societal engagement initiatives have been established at national and European Union (EU) level. Prominent examples include the public dialogue organized by the British Biotechnology and Biological Sciences Research Council (BBSRC) (Bhattachary et al., 2010), a variety of science cafés (Navid and Einsiedel, 2012), and the EU-FP7 project SYNENERGENE.

Against this background, we are interested in the meaning that is attributed to synbio in societal engagement processes. The central question for our analysis is whether synbio produces new pictures, enforces new narratives, releases new conflicts – and which established meanings from previous technology controversies (notably biotechnologies) are reproduced. With this analysis, we aim to contribute to the broader debate on the relationship between publics and technologies in the context of RRI. Conceptually, we draw on notions of frames and framing in media studies and policy analysis (‘The power of frames’ section). Empirically our discussion is based on the observation and analysis of 10 engagement events that were organized in the context of the EU-FP7 project SYNENERGENE (‘Cases and methods’ section). We present six distinct frames and related narratives that are established in deliberations on synbio (‘Frames in the emerging synbio discourse’ section). Overall, our analysis shows that frames that problematize the technology become less prominent in favour of new technology optimism and the retraction to abstract meta-debates (‘Conclusion’ section).

## 2. The power of frames

Even though the concepts of frame and framing have been taken up by wide range of academic disciplines since the 1990s (van Hulst and Yanow, 2014), the ongoing social-scientific debate has strongly been influenced by *media theory* and *communication studies* with their focus on ‘media frames’ or ‘news frames’. From this perspective, frames are cognitive schemata which help the reader to come to terms with complex issues ‘by lending greater weight to certain considerations and arguments over others’ (Nisbet, 2010: 44); they are ‘organizing devices’ (Nisbet and Lewenstein, 2002: 361) for the presentation of complex issues in a way that makes them accessible to a broader public (Scheufele and Tewksbury, 2006). As media frames have often been investigated with regard to their effects on public attitudes, empirical studies focused on how interest groups influence frame-building processes (Callaghan and Schnell, 2001). Powerful actors are suspected of choosing among frames reflectively and consciously in order to promote their interests (Chong and Druckman, 2007).

In the field of *public policy studies*, frames are conceived as interpretative schemes playing a prominent role in political controversies (van Hulst and Yanow, 2014). Political controversies emerge when incompatible policy frames compete to define a policy problem and to prescribe respective courses of action (Schön and Rein, 1994). In contrast to policy disagreements, gridlocked political controversies cannot be solved by reference to facts, but by a process of ‘frame reflection’ (Schön and Rein, 1994). This means changing the level of conflict resolution.

Scholars in Science and Technology Studies (STS) have often emphasized the powerful character of frames (Jasanoff, 2003; Levidow and Carr, 2007; Wynne, 2007). Frames reproduce order (identities, relationships, worldviews) by providing certain modes of perception and interpretation. They represent a form of ‘symbolic power’ (Bourdieu, 1989). This power is anything but gentle, because it is capable of making the current order and ontology, habitual identities, and relationships appear essential. To problematize technologies, actors necessarily refer to arguments and keywords considered highly relevant (such as ‘risky’ or ‘morally questionable’). Thus, the individual interpretation of a technology has its starting point in influential frames elaborated in the mass media, by the political elite and social movements, in former technology conflicts, and so on. However, this does not mean that frames are just passively adopted and that laymen’s interpretations are just the echo of experts. Rather, frames have a flexible structure, that is, we performatively update and modify these schemata. For this reason, it is important to distinguish between *frames*, that is, pre-existing schemes of interpretation, and *framing*, that is, the processes through which actors seek to impose interpretations and order upon an ambiguous social world (Hajer and Laws, 2006).

Concerning the ontological status of frames, both policy studies and STS emphasize that frames are neither stable things hidden in texts nor simply instruments provided by mass media in order to cope with the complex world. Contrary to a cognitivist reading, frames must first be considered plausible and legitimate in order to become influential in collective sense-making. Frames maintain their legitimacy because they draw upon historically handed down and culturally mediated narratives and interpretations as Macnaghten et al. (2015) have pointed out. Accordingly, framing is a tacit process. In contrast to some approaches from the fields of media theory, we assume that in technology conflicts the opponents mostly do not deliberately choose between alternative framings. Most often the opponents are not aware why they are referring to certain frames. This implicitness gives conflicts a focus; without a commonly shared frame, conflicts would meander aimlessly and constantly change shape (i.e. topics, arguments, keywords) so that opponents would not be able to refer to each other in a meaningful way. So, on the one hand, the power effects of framing are productive, because with reference to established frames, contradiction and dissent can be made visible and communicable. On the other hand, frames are also restrictive, because the dissidents are fixed on certain themes and argumentation paths and cannot simply intervene from an imaginary ‘outside’.

Regarding controversies over environmental and technology issues, several frame typologies – often referring to Gamson and Modigliani’s (1989) analysis of the US media discourse on nuclear power – have been developed. Even if it is not possible in the context of our article to discuss the multitude of frame typologies in further detail, we find recurring frames throughout diverse analysis: scientific progress; societal benefits; risks, uncertainty and runaway science; ethics/morality; governance and public accountability; economic prospects; and public opinion and conflict (Davis and Russ, 2015; Maeseele and Schuurman, 2008; Nisbet, 2009; Nisbet et al., 2003; Scholte et al., 2013). In previous debates on biotechnology, a handful of frames played a crucial role, among them ethics, risks, and economics (Bogner and Torgersen, 2015). For example, negotiating whether the technology option at stake is morally right or wrong the opponents (im- or explicitly) refer to an ethics frame. Important keywords and metaphors are autonomy, beneficence, human life protection, or playing god. Consequently, frames provide umbrellas under which arguments both for and against the technology can be brought forward and negotiated. In concrete terms, this means that one can be for or against stem cell research, but the justification of one’s own position must be of an ethical nature, provided that ethics has been established as a dominant frame or ‘master frame’ (Dahinden, 2002) in the debate.

## 3. Cases and methods

Our analysis of the framing of synbio in societal engagement processes builds on the project SYNENERGENE (Responsible Research and Innovation in Synthetic Biology)^1^ that was funded by the European Commission under the 7th Framework Programme (FP7). The consortium was coordinated by Karlsruhe Institute of Technology (KIT) and consisted of 27 partners in 16 countries, including research organizations, companies, think-tanks, a network of science centres and museums, an art society, science journalists, a theatre, and civil society organizations. The project aimed at contributing to Responsible Research and Innovation in synbio by initiating public dialogue and mutual learning processes among stakeholders from science, industry, civil society, education, arts, and the broader public. In the course of the project (2013–2017), a variety of engagement events were organized across Europe.

We, the authors, were official partners in the project with the task to analyse public engagement events with a view to the process of framing and the character of participants’ involvement. Our analysis is based on the observation of 10 engagement events (see Table 1), which we selected along the following considerations: First, the events present a variety in terms of target groups, that is, we included events that mainly addressed stakeholders and experts (E1, E2, E3, E7, E8) and events that addressed the wider public and lay audiences (E2, E4, E5, E6, E8, E9, E10). Several events addressed multiple actor groups and combined various formats and therefore are listed twice. Second, the events represent well-established engagement formats, including stakeholder workshops (E1, E2, E3, E7, E8), science cafés (E8, E9), and public panel discussions (E5, E6), as well as novel formats such as theatre performances (E4, E5, E8) and film screenings (E2, E8). In addition, we included an online debate with high school students (E10) as an illustration of digital engagement formats.^2^ Third, language was a constraining factor in the selection of events. With the exemption of one event (E7), we observed events that were held in English or German to ensure an in-depth analysis of the debates. The Dutch stakeholder workshop (E7) was observed by a colleague from Rathenau Instituut. Moreover, we conducted an interview with one of the organizers of the event.

**Table 1.** Analysed events.

No.	Event title	Date & place	Format and interactional character	Participants	Organizers
1	Workshop “Responsible Research and Innovation in Synthetic Biology”	23–25 June 2014, Darmstadt, Germany	Stakeholder workshop, combining presentations and group discussions	~50 stakeholders and experts	Institute for Technology Assessment and Systems Analysis (ITAS) at the Karlsruhe Institute of Technology (KIT); Technical University Darmstadt; Schader Stiftung
2	BioFiction Science Art Film Festival	23–25 October 2014, Vienna, Austria	Film festival + accompanying activities: conference, workshop on DIY-biology, a resident artist’s work in progress, dancing performance	80–120 students, local public, experts, stakeholders	Biofaction (Research and Science Communication Company, Vienna)
3	Regional Stakeholder Workshop “Perspectives and Key Issues in Synthetic Biology: Opportunities, Risks and Policies”	18–19 June 2015, Ljubljana, Slovenia	Stakeholder workshop, combining presentations, and group discussions	40 stakeholders and experts	University of Ljubljana
4	Science on Stage: “Supernova SynBio Hack Mack”	28 June 2015, Karlsruhe, Germany	Theatre play including Q&A session with audience	~40 local public, 2 experts	ITAS/KIT in collaboration with the Centre for Cultural and General Studies at the Karlsruhe Institute of Technology (ZAK/KIT) & Badische Staatstheater
5	Thematic Congress “Re-engineering Life or do we want to be eternal?”	3–4 July 2015, Freiburg, Germany	Combination of panel discussions, theatrical performances, speed dating with experts, film presentation, acoustic and a graphic performance	30–60 students, local public, experts	Theatre Freiburg; University of Freiburg; Université Paris 1 Panthéon-Sorbonne
6	Public Outreach Event “Talks of the Town: From Karl Drais to Synthetic Biology”	16 September 2015, Karlsruhe, Germany	Panel discussion	130 local public, 3 experts	ITAS/ KIT in collaboration with ZAK/ KIT
7	Workshop “Antimicrobial Resistance and Synthetic Biology”	23–24 March 2016, Amsterdam, The Netherlands	Stakeholder workshop, combining presentations and scenario workshops	14–19 stakeholders, experts	Rathenau Instituut of the Royal Netherlands Academy of Arts and Sciences
8	Synthetic Biology Forum “Synthetic Biology–Visions of the Future”	24–25 June 2016, Amsterdam, The Netherlands	1st day: stakeholder conference combining presentations and panel discussions 2nd day: public outreach with science café, interactive theatre performance, workshop, panel debate, film screening	80–180 experts, stakeholders, local public	Rathenau Instituut in collaboration with SYNENERGENE partners
9	Science Café “Altering plants, microbes and people”	23 November 2016, Bristol, United Kingdom	Input by scientists, extended Q&A with audience	~50 local public, 6 scientists	University of Bristol (BrisSynbio Centre for Public Engagement)
10	SYNENERGENE Online Debate	May–June 2017	Online platform with introductory texts and videos, opportunities to post comments and questions, answered by experts	18 high school students, 3 experts	Zebralog; University of Bristol; ITAS/KIT

Our observation was guided by a semi-standardized protocol that was adapted to the specifics of the single events (see Supplemental Material). To enable the later frame analysis, we logged all verbal contributions (e.g. presentations, comments, questions) in their sequences. We did not yet assign frames during the observations but wrote down what was said, by whom and how (e.g. explanatory, moralizing, emotional). Regarding interactional aspects, we took notes of the settings of the events (e.g. the seating of participants), the procedure (in terms of agenda and phases), the involved actors (e.g. numbers, age, and gender), and their roles (e.g. experts, audience, or moderators). We further assessed the communicative character of the debate (i.e. whether it was contentious or consensual). In regard to videos and theatre performances, we transcribed the contents (what was it about, which arguments were put forward) as well as their dramaturgy (how the content was presented) and how the audience was involved.

The subsequent analysis of the framing of synbio combined deductive and inductive strategies of qualitative frame analysis (de Vreese, 2005). In a first step, we closely read the protocols and prepared descriptive summaries of the main themes and issues (e.g. synbio’s applications in health, Do-it-Yourself-biology (DIY-biology), or risk management) of the single events. Subsequently, we analysed the protocols for framing devices, that is, keywords, catch phrases, anchor examples, metaphors, and arguments (de Vreese, 2005; Pan and Kosicki, 1993; van Gorp, 2007, 2010). Renowned frame typologies (see ‘The power of frames’ section) served as sensitizing concepts for the identification of framing devices and the corresponding assignment to frames. For example, the metaphor ‘designer babies’ is a prominent framing device that has accompanied biotechnology debates for a long time. Based on recurring topics, arguments, comparators, and metaphors within the frames, we reconstructed key narratives that characterize specific frames (see Table 2). Although we based our analysis on previous research on environmental and technology frames, we aimed at remaining open towards new frames or novel manifestations of renowned frames. Therefore, all those passages in the protocols whose meaning remained open with regard to underlying frames were subjected to a further round of analysis in which we identified additional framing devices and narratives. For example, the issue of DIY-biology and respective imaginations of open science (or epistemic democratization) were new additions to the biotechnology discourse that signified a reconfiguration of the traditional economy frame (see below).

**Table 2.** Frames in the synbio discourse.

Frame	Core narratives	Frame-signifying elements (key issues, examples, metaphors, arguments)
Science	(a) Synbio as (continuous) scientific progress (b) Synbio as a new way of doing biology	Crispr/Cas 9, gene drives, precision, speed Recreation, redesign, engineering
Social progress	(a) Synbio as a key for solving societal challenges (b) Synbio as the newest techno-hype and technofix	Grand challenges: bioeconomy, environment, health, food Artemisinin, past techno-hypes, questionable realizations, social innovations, framing
Risks and control	(a) Synbio as risks concerning biosafety and biosecurity (b) The risks are controllable (c) The risks are uncontrollable	Lab accidents, criminal or terrorist misuse, DIY-biology Standards, assessment procedures, risk management, past experiences Gene drives, mammoth, lack of knowledge, irreversibility, DIY-biology
Ethics	(a) Ethics is key to RRI (b) Imperative to innovate for a better world (c) Uncontrollable risks are morally not acceptable (d) Are we allowed to do what we are able to do?	Ethics as a driver of innovations, societal benefits and values (autonomy, privacy etc.) Envisioned applications for bioeconomy, environment, health, food; urgency Gene drives, eradication of mosquitoes Playing god, George Church, designer babies
Economics	(a) Synbio as manifestation of neoliberal and capitalist appropriation of nature (b) DIY-biology as a counter-model to capitalism: open, bottom-up and non-hierarchical	Monopolization, big industries, patenting of life, exploitation of nature Bio-hackers, DIY-communities, iGEM, open participation, open access
Governance	(a) Synbio regulation and risk assessment (b) Synbio as a case for RRI (c) Need for public engagement	Comparison to GMO regulation, tentative governance What does RRI mean, implementation in R&I policies and practice Alignment with societal values, deficit model

RRI: Responsible Research and Innovation; GMO: genetically modified organisms.

During the interpretation and reconstruction of the frames, we combined the content analysis with the analysis of our observations of the interactions in the events, that is, we reflected what has been said against who said it and how it was said. Thus, in the following section, we do not only describe the content of the frames but also indicate how particular issues were introduced, which actors promoted key arguments and whether competing narratives emerged. To ensure robustness of the results, the analyses were undertaken by two analysts who first analysed the transcripts and protocols separately and then jointly discussed difficult or marginal passages, issues, and framing devices.

## 4. Frames in the emerging synbio discourse

In our frame analysis, the aspect of social interaction among stakeholders, laypeople, and experts plays a crucial role. Since the analysed events varied widely in formats, settings, target groups, and attendance, they facilitated distinct ways in which frames could be invoked, adapted, and renegotiated. In terms of thematic orientation, the observed stakeholder workshops, with the exemption of the workshop on Antimicrobial Resistance (AMR) (E7), addressed synbio with a broad focus, for example in the context of RRI (E1, E3), risks, opportunities, and policies (E3) or synbio visions (E8). Conference-style presentations and panel discussions, involving experts and stakeholders, were common elements in all workshops, providing information and specific perspectives on synbio as inputs for further debates. In addition, the stakeholder workshops allotted ample time for exchange of experiences and perspectives among participants in small group discussions or scenario exercises (E7). Generally, stakeholders and experts were engaged on equal footing and the debates were characterized by a rational and calm nature with only few fundamental controversies. In several cases (E1, E2, E3), we observed debates that revolved around emerging technologies in general, rather than specifically around synbio.

Similarly, most public outreach events introduced synbio generically as well. In terms of engagement formats, the public events included formats such as science cafés (E8, E9), public panel discussions (E6), or an online debate (E10) as well as formats making use of genres of imagination and art such as films or theatre performances (E2, E4, E5, E8). Overall, we observed a strong emphasis on educating the audience. In many cases, experts were prominently involved, providing information and explanations in introductory statements, as panel participants or respondents throughout the events (E4, E6, E9, E10). Also, artistic formats were used in an educational way. For example, the Karlsruhe theatre play (E4) was written and commented by scientists, combining information with synbio visions and entertainment. The emphasis on information in the beginning of most events strongly influenced the character of the subsequent exchange between experts and participants. While in most events, ample time was dedicated to the involvement of the audience, the interaction was often limited to single audience members posing questions and experts providing factual clarification or their perspective (E4, E9, E10). In these settings, we hardly observed the emergence of a genuine debate. Consequently, the discussions remained rather ‘scientifically-minded’, switching in a Questions and Answers (Q&A) format from one topic to the next. Film and theatre performances that emerged from the engagement of artists with synbio (E2, E5) introduced perspectives different from the academic discourse and served the stimulation of the imagination, for example by confronting participants with ethical questions and future visions (e.g. in E5, the re-awakening of the mammoth was theatrically staged). Yet, the performances were hardly used to induce a debate with or among audiences. Few participation tools allowed for a more interactive debate between experts, stakeholders, and publics, including a speed-dating with experts (E5), a fishbowl round involving audience members (E6), or an interactive theatre debate (E8).

This brief sketch of interaction settings already points to key framing features of the observed events. The emphasis on information provides experts and organizers a prominent role in setting the agenda (and therewith introducing the relevant frames for the debate), particularly when the issue is largely unknown. The absence of actors with clear positions and interests might indicate an openness of the debate in terms of frames. Against the background of these different interaction settings, we observed the introduction, negotiation, and challenging of six distinct frames: (a) synbio as science, (b) synbio as social progress, (c) synbio as an issue of risks and control, (d) synbio as an ethical question, (e) synbio in economic context, and (f) the governance of synbio (see Table 2). Within these frames, we reconstructed various, at times contradicting narratives, each telling a different story about what synbio is, which opportunities and challenges synbio brings, and how they should be addressed by science, politics, and society.

### Science frame: Synbio as a new technoscience

In all events, synbio was introduced as a scientific endeavour that involves scientific advances, new research practices as well as new venues of research such as community labs or garages. The science frame was invoked, whenever experts or moderators gave scientific and technical explanations, introduced specific research areas, or addressed knowledge gaps as well as scientific and technical challenges. The science frame was invigorated whenever audience members asked for technical clarifications. Prominent subjects of explanations included the techniques CRISPR/Cas9 and gene drives. Explanations often compared synbio to practices of traditional biotechnology, characterizing synbio as a more precise and faster technique (E8). While in this narrative, synbio is scientific progress in a linear and continuous sense, scientists also frequently introduced synbio as a fundamentally new science, breaking with traditional biological research (E5). The narrative went like this: ‘conventional biology was predominantly about analysing and understanding nature. Synbio is about re-creating and re-designing living things by combining biology with principles of engineering and computer sciences’ (E5, E8). The current status of synbio was largely characterized as changing living things, while the notion of creating life pointed towards future visions of synbio. Prominent examples within the ‘re-creating life narrative’ included Craig Venter’s synthetic bacteria (E5, E9) and George Church’s plan to resurrect the mammoth (E5). At times, experts criticized the reductionist understanding of science and nature that underlie the visions of controlling nature and evolution (E5).

### Social progress frame: Synbio tackling grand societal challenges

The social progress frame was the most frequent and consistent frame across all events, centring on the question whether and how synbio could contribute to a better life. The following narrative dominated the frame: *‘Synbio is a powerful technique for addressing current Grand Societal Challenges*^3^ *and therewith has a high potential to contribute to a better and more sustainable future’.* Moderators, experts, and stakeholders frequently substantiated this narrative by linking (mostly envisioned) applications of synbio to current societal challenges, notably in the areas of environment, health, and food. For example, a recurring vision for realizing the bioeconomy was the application of synbio techniques to genetically alter algae to produce biofuels as a contribution to the transition to renewable energy production. Synbio applications in the health sector constituted a further sturdy pillar of the ‘solution narrative’. The introduction of the topic health in the online debate (E10) illustrates the associated high expectations:

An increased understanding of human health at the molecular level, combined with increased options for manipulating genomes is driving a hopeful future for improvements in medicine. For example, synthetic biology and genome editing could offer new forms of gene and gene-based therapies against HIV and cancer. Infectious diseases such as malaria or zika could be fought against by genetically manipulating their carriers, such as mosquitoes, using so-called gene drives.

The synthetic production of the malaria vaccine Artemisinin played a central role as one of the few synbio applications in medicine that already existed. Medium-term visions prominently centred on synbio’s potential to address the problem of AMR and long-term visions included promises of curing cancer, synthetic organs, and personalized medicine. As these exemplary visions illustrate, within the social progress frame synbio largely symbolized optimism, that is, the promise of a better life and more sustainable future. Synbio was presented as a solution, and at times even salvation, to many current problems. This narrative was supported by synbio scientists and students that explained the purpose of their research, but also by organizers and moderators of the events that, in the context of RRI, linked synbio to current Grand Challenges.

Although the ‘solution narrative’ was pervasive, it was not uncontested. To avoid a technology bias, moderators, organizers, and experts introduced warnings against inflationary expectations, references to the (limited) state of the technique, the gaps between the promises and actual progress as well as the meagre success of synbio applications so far (again Artemisinin served as a prominent example). Moderators, stakeholders, and audience members occasionally referred to unredeemed techno-promises in the past (such as nuclear power or robotic technology) to warn against credulous beliefs in the promising visions. Representatives of critical non-governmental organizations (NGOs) challenged the ‘solution narrative’ by deconstructing it as a technofix, which was strikingly summarized in the Amsterdam Forum (E8) with the phrase ‘when you have a hammer, everything looks like a nail’. In this alternative narrative, synbio was looking for challenges in order to legitimize research rather than challenges waiting for synbio as a solution. By the fixation on the technology (synbio), the causes of the manifold societal challenges were obscured and other, less risky and controversial, solutions were ignored. Hence, critical stakeholders (and occasionally citizens) re-defined the social progress frame by pointing towards the causes of current challenges and alternative technological and social solutions.

### Risk frame: Controlling biosafety and biosecurity risks

The risk frame was frequently addressed by organizers and moderators. Yet it was only generically taken up by stakeholders or citizens, stating that there might be risks involved, yet without further substantiation what these risks might be. When risks were addressed in a more substantial manner, two narratives were present – biosafety and biosecurity. The ‘biosafety narrative’ entailed concerns about accidents in laboratories which could result in the unintentional release of genetically modified organisms (GMO) in the environment. In addition, the potentially negative environmental consequences of the intentional release of GMOs, for example, the eradication of species (such as mosquitoes) for ecosystems, served as a counter-narrative to the ‘solution narrative’. The ‘biosecurity narrative’ revolved around the intentional misuse of the technology by criminals or terrorists, that is, ‘bad guys doing bad things’ (E9). The most concrete example was the potential revitalization of the Spanish flue. DIY-biology was a prominent theme in both, the ‘biosafety and biosecurity narrative’. With the technology (synbio), respective materials, and machines becoming easily available for anyone, concerns about the safety standards outside established laboratories in academia and industry raised particular concerns, as the following statement, issued by a student in the online debate (E10), illustrates:

Why do people have to try such dangerous stuff at home in their own garage with the risk, that they could create something new, something very dangerous for everybody? Why is it possible that people do this and is it really necessary that this DIY-Biology is getting supported? What if somebody is creating a new dangerous bacterium?

Concerning the control of these risks, two narratives competed. On the one side, synbio scientists presented the risks as manageable by adequate risk assessment, safety and security standards, and governance regimes. References to scientific and laboratory standards, the contained environment of synbio experiments, the building in of safety checks, high regulatory standards, existing oversight mechanisms, and the education and training of scientists served to ensure that risks could be controlled. In regard to DIY-biology, the actual possibilities of producing and releasing dangerous organisms were presented as negligible. In addition, concerns were allayed by normalizing the risks as the following statement by a synbio scientist illustrates (E9): ‘You could already now build a biological weapon, if you wanted, there is no shortage of organisms with which you could do that’ with another scientist adding: ‘[. . .] it doesn’t happen, so I find that encouraging’.

The ‘control narrative’ was countered by NGO representatives and partly social scientists with references to existing knowledge gaps (e.g. about gene flow or the definition of acceptable amounts of escape). The adequacy of existing safety standards against the release of modified genetic elements in the environment was questioned and scepticism about the capability of science to anticipate and deal with the risks was raised (E1, E2). At times, scepticism about the controllability of risks turned into the image of ‘runaway science’. The gene drive technology with the palpable application of the eradication of mosquitoes served as a central example for the dangers of releasing something into nature that cannot be contained, controlled, or reversed. Comparisons to the release of rabbits in Australia or the dangers of the hydrogen-bomb further substantiated how control over technologies is easily lost (E9). The notion of synbio as runaway science was accompanied by a fatalistic tone: ‘there is nothing you can do about it’ (E9).

### Ethics frame: Ethics as a driver for innovation

RRI implies an increasing importance of ethics as a means of shaping innovation responsibly and proactively (Owen et al., 2012; von Schomberg, 2013). To be indicated as responsible, research and innovation should contribute to finding sustainable solutions for societal challenges. Against this background, the discussion of synbio being ethically right or wrong within SYNENERGENE events frequently referred to the expected benefits of synbio applications on the one side and the risks and unintended impacts on the other side, and therewith strongly interlinked with the social progress and risk frames. As a consequence of the dominance of the ‘solution narrative’, the benefits were frequently presented as outweighing the risks. The close link between technological innovations through synbio and social progress at times invoked a high necessity to advance synbio research and innovation (E9, E10). The lack of time (and options) in regard to the manifold societal challenges served as a legitimization or even moral imperative for further developing and quickly marketing the technology. To hinder innovations in synbio would be irresponsible in the face of the urgency of societal challenges such as climate change or the severity of illnesses. Innovation in this context is not a choice but a moral duty to current and future generations. On the other side, the severity and uncontrollability of potential risks of synbio was used to argue for precaution or even moratorium of particular synbio innovations. The most outstanding example was the use of gene drives for the eradication of mosquitoes (E8). Thus, ethics under RRI predominantly follows a consequentialist argumentation. In contrast, classical deontological questions such as ‘are we allowed to play god?’ or ‘are we allowed to interfere in nature to create life?’ were less frequently raised. The metaphor of ‘playing god’ was invoked in artistic formats such as theatre performances and films (E2, E5) to convey strong ethical considerations and explore the ethical limits of synbio.

As a further consequence of the accentuation of ethics through the guiding RRI notion, ethics was frequently addressed in a generic manner rather than in concrete reference to synbio. Rather than emerging from the views, values, and concerns of participants, ethics became a compulsory default element to address. Questions of the universality of ethical standards versus value pluralism in the era of globalization were raised generically and, in several events, philosophers and social scientists criticized the absence of the discussion of ethical aspects, yet without introducing concrete ethical questions (E1, E2).

### Economics frame: Criticizing bio-capitalism

The economics frame was a constant throughout the events, yet in a different configuration than observed in previous technology debates (Bogner and Torgersen, 2015). The classical narrative of technological innovations as a driver for economic growth and competitiveness was only marginally addressed. Instead, questions concerning the access to the technology, patenting of nature, and the control of synbio were integrated in two different economic narratives.

In line with a critical economic narrative, NGOs and occasionally social scientists and citizens depicted synbio as a manifestation of degenerated capitalism and neo-liberalism. In analogy to agro-biotechnology, it was assumed that synbio would ultimately be appropriated by few multinational industrial players, reinforcing current inequalities and power structures. Monsanto served as a paragon for the monopolization and industrial exploitation of nature that were expected to continue with synbio. Private ownership for biological entities (‘privatizing life’) would only serve big industrial players and imply further dangers for the economies of developing countries (E2). NGO representatives substantiated such claims by highlighting the already negative impacts of synbio products (such as Artemisinin or synthetic vanilla) on farmers in developing countries (E8). In this context, the discussion about intellectual property rights was led critically: the patenting of nature was seen as a perversion of the neo-liberal project that aims at the instrumentalization of life for economic reasons.

Sharing the fundamental critique on the economic exploitation of biotechnology, representatives of DIY-biology offered a more optimistic outlook by framing synbio as an opportunity for more democratic and community-driven forms of innovation. DIY-biology in community labs, the biohacker community, and iGEM^4^ were presented as bottom-up initiatives that manifest a counter-model to the hierarchical and protracted character of established academia and the exploitation of nature by big industries. Such initiatives were presented as open to playing and tinkering but also striving for societal advances (e.g. in health and environment) without economic interests. The attempts of biohacker communities to address the AMR challenge were repeatedly referenced (E8). The key principle of openness referred to the participants as well as to the access to the technology (E10). The novelty with synbio is that everybody is entitled to do it, undergraduates as well as laypeople may become active scientists even if they lack academic degrees (‘It’s biology that you do yourself. Yes you!’, from the introductory text in the online debate, E10). The results of research and innovation would be available for and benefit all and consequently counter capitalist accumulation tendencies.

### Governance frame: Synbio as a test case for Responsible Research and Innovation

The question ‘how to govern research and innovation?’ played a central role throughout the observed events. A prominent issue revolved around the question whether current regulations were sufficient with regard to biosafety and biosecurity (see risk frame). The dominating conclusion was that it is currently too early to know what regulatory needs would emerge and that existing regulative frameworks for gene technology were appropriate and sufficient for the moment. Based on visions of ever faster developments in synbio, however, the need for an adaptive governance approach, including new tools and methodologies of risk assessment and management, was anticipated.

Moreover, as observed for the ethics and risk frames, the governance frame was predominantly invoked at the meta-level. RRI played a prominent role in such generic debates: How to interpret the concept and how to implement it in current research and innovation practices? How to responsibly govern emerging technologies with regard to possible risks, safety and security issues, and the demand for public participation? In particular, the question of the added value and design of societal engagement in research and innovation served as a focal point for debates on RRI. Frequently, the importance of societal engagement and dialogue was emphasized, though along different rationales. On the one side, organizers of the events as well as social scientists promoted public engagement as an essential part of RRI, informing or even influencing research and innovation activities and securing their alignment with societal values. On the other side, scientists understood engagement as an opportunity to inform the public and gain acceptance for new technosciences (E3, E9). In this context, synbio scientists at times invoked the well-known deficit model: Cacophony, misinformation, and laypeople’s ignorance were seen as major obstacles for a genuine debate on and the advancement of synbio, as was observed in the case of GMOs. Against this background of ‘the problematic public’ enhancing scientific literacy of the public emerged as a main governance challenge (E3, E9).

## 5. Conclusion

How does the public debate on synthetic biology unfold in the context of invited participation? At a glance, our analysis suggests that established frames of previous technology debates (ethics, risk, social and scientific progress, economics, and governance) are reproduced. Yet, the in-depth analysis of the narratives, issues, and arguments that constitute these six frames implies significant reconfigurations. Thus, we find that the frames that traditionally served to problematize biotechnology, that is, ethics, risks, and economics (Bogner and Torgersen, 2015), become less pronounced, implying a shift away from the traditional notion of ‘technology as conflict’ (Torgersen and Schmidt, 2013). Instead, there are three conspicuous features of the framing of synbio: (1) the tendency towards meta-frames, (2) technology optimism, and (3) the ambiguous role of DIY-biology. We concludingly reflect, in how far these features result from the interaction quality of the events or are symptomatic of broader tendencies in the public framing of technosciences.

The first observation striking is the high abstraction of debates. Particularly when it comes to questions of ethics, risks, or governance, the debates frequently remain on a generic level with no or only minimal reference to synbio. In the extreme, participants do not adopt ethical positions or point to potential risks at all, but rather emphasize the general value of ethical reflection for technology governance. Even if ethical questions are touched upon, participants frequently engage in a kind of standardized ethics patterns for new and emerging science and technology (so-called NEST-ethics), that is, they make statements about technological progress, its desirability and potential impacts that could apply to any emerging technology (Swierstra and Rip, 2007: 4). This observation may be explained by the unfamiliarity of synbio among participants, a lack of concrete interests and concerns as well as the dominance of social scientists in many events. However, as other studies have demonstrated (see Betten et al., 2017; Swierstra and Rip, 2007), the recourse to NEST-ethics is not unique to the observed events, but has become a dominant interpretive repertoire and cultural toolkit that is drawn upon whenever new techno-debates emerge (Macnaghten et al., 2015; Swierstra and Rip, 2007).

Second, we observe a dominance of the social progress frame with a predominantly optimistic ‘solution narrative’. Synbio is associated with high expectations of conquering major societal challenges, such as clean energy, health, or environmental protection. In the observed events, the introduction of envisioned applications made synbio more tangible for audiences, thereby moderating the tendency towards generic debates at the risk of introducing a technology bias. This dominance of the social progress frame is not specific for the analysed engagement context and respective formats. A range of media studies conclude that synbio is predominantly presented in a positive light, giving more focus to potential benefits than risks (Ancillotti et al., 2016). A study by Schmidt et al. (2015) suggests that also artistic views on synbio strongly promote the idea of ‘technology as progress’. Notably, the embedding of the project in the RRI discourse does not challenge, but rather reinforces this technology optimism. This observation empirically supports interpretations of other scholars (de Saille, 2015; Delvenne, 2017) stating that RRI emphasizes technological innovations as the main solution to economic and societal challenges. Yet, our analysis has shown that this framing can also be actively challenged, in our case by giving NGOs a prominent role in engagement events.

The (new) techno-optimism is further reflected in the (self)-presentation of the DIY-biology movement. Since the project SYNENERGENE aimed at engaging DIY-activists as well as iGEM students, this new movement gained more prominence in the events than in other engagement initiatives or media studies. Regarding the social progress frame, the activities and visions of DIY-biologists and iGEM students easily align with the expectations raised by scientists and therewith reinforce the dominant ‘solution narrative’. From an institutional point of view, however, DIY-biology calls for a counter-model to the established entanglements between basic research, biotechnology and industry and, thus, represents a new social movement in the name of open science (Delfanti, 2013). Yet, as much as DIY-biology implies optimism and change, it is also a prominent subject of fears in the public discourse. Within the risk frame, the picture of DIY-biology is strongly associated with uncontrollability (the hackers in the garage) and the notion of runaway science (accidents and misuse). In this frame, the picture of DIY-biology is significantly transformed, from a vibrant and engaged youth movement to a ‘group of concern’ (Frow, 2017).

The analysed public outreach events served as a test-bed for societal engagement on emerging technologies in general and synbio in particular. Only when the public is given a legitimate place in technology policy, as Habermas (1970) argued, does it becomes possible to problematize technologies. Consequently, the public’s involvement indicates that technology in general has become questionable. However, as we have shown, public engagement in the context of RRI may turn into a well-organized, calm, and sterile expert-like debate. Rather than defining and reframing the debate on synbio and giving voice to the concerns, values and interests of various publics, public engagement events are at risk of reproducing dominant narratives and frames that emerged in other contexts (academia, media, politics, industry). Against this background, our analysis may help engagement practitioners to identify neuralgic points and critical dynamics in engagement processes through which particular framings become dominant or may be questioned. As our analysis has shown, it is important to keep the discussion specific and relevant to avoid meta-debates. In addition, organizers could make use of the variety of frames to induce a multi-facetted debate. By openly introducing different framings of synbio, a reflection of the participants on their own positions and views can be encouraged. Against the background of an almost non-existing public debate on synbio and an assumed lack of synbio knowledge on the side of the public, organizers of public dialogue events often strongly focus on information at the expense of two-way interaction, thereby reinforcing the deficit model. In consequence, the debates frequently remain academic, rational, and uni-directional. Yet, leaving room for emotional and affective reactions as well as for controversies is an important factor for a lively and successful public debate. Artistic engagement tools, such as films and theatre plays, could offer opportunities for more emotional and creative views on synbio. In conclusion, societal engagement in the context of RRI should not only aim at involving an increased number of participants but also at enabling various modes of arguing and reasoning. Only then, the added value of societal engagement materializes: the demonstration of diversity, breadth, and dissent that may lead to new perspectives and options in research, technology, and innovation governance.

## Supplemental Material

framing_synbio_observation_protocol – Supplemental material for Let’s (not) talk about synthetic biology: Framing an emerging technology in public and stakeholder dialogues Click here for additional data file. Supplemental material, framing_synbio_observation_protocol for Let’s (not) talk about synthetic biology: Framing an emerging technology in public and stakeholder dialogues by Anja Bauer and Alexander Bogner in Public Understanding of Science

## Notes

1. See the archived project website https://www.synenergene.eu/index.html (last accessed 3 December 2019)

2. The online debate was introduced and discussed in selected schools. On the platform, students were introduced to six specific issues around synbio and could pose questions or comments that were answered by experts (yet there was also the possibility given to react to other students’ comments). The students were informed that we would analyse the contents of the debate, preserving their anonymity.

3. Prominently, the European Commission has defined seven societal challenges in the context of the research programme Horizon 2020: (1) health, demographic change and well-being; (2) food security, sustainable agriculture and forestry, marine and maritime and inland water research, and the bioeconomy, (3) secure, clean and efficient energy; (4) smart, green and integrated transport, (5) climate action, environment, resource efficiency and raw materials; (6) Europe in a changing world – inclusive, innovative and reflective societies; (7) secure societies – protecting freedom and security of Europe and its citizens (see https://ec.europa.eu/programmes/horizon2020/en/h2020-section/societal-challenges, last accessed 3 December 2019).

4. iGEM stands for international Genetically Engineered Machine competition. In this competition, students work together in multidisciplinary teams in the field of synthetic biology ‘using interchangeable biological parts and standard molecular biology techniques’ (see https://igem.org/Main_Page, last accessed 17 December 2019).

## References

[bibr1-0963662520907255] Ancillotti M Rerimassie V Seitz SB Steurer W (2016) An update of public perceptions of synthetic biology: Still undecided? Nanoethics 10(3): 309–325.

[bibr2-0963662520907255] Betten AW Broerse JEW Kupper F (2017) Dynamics of problem setting and framing in citizen discussions on synthetic biology. Public Understanding of Science 27(3): 294–309. 28597721 10.1177/0963662517712207 PMC5843019

[bibr3-0963662520907255] Bhattachary D Calitz JP Hunter A (2010) Synthetic biology dialogue report. Available at: https://bbsrc.ukri.org/documents/1006-synthetic-biology-dialogue-pdf/

[bibr4-0963662520907255] Bijker WE (2009) Social construction of technology. In: Friis JKBO Pedersen SA Hendricks VF (eds) A Companion to the Philosophy of Technology. Chichester: Wiley Blackwell, pp. 88–94.

[bibr5-0963662520907255] Bogner A Torgersen H (2015) Different ways of problematising biotechnology: And what it means for technology governance. Public Understanding of Science 24(5): 516–532. 24966166 10.1177/0963662514539074

[bibr6-0963662520907255] Bourdieu P (1989) Social space and symbolic power. Sociological Theory 7(1): 14–25.

[bibr7-0963662520907255] Callaghan F Schnell K (2001) Assessing the democratic debate: How the news media frame elite policy discourse. Political Communication 18(2): 183–213.

[bibr8-0963662520907255] Chong D Druckman JN (2007) Framing public opinion in competitive democracies. American Political Science Review 101(4): 637–655.

[bibr9-0963662520907255] Dahinden U (2002) Biotechnology in Switzerland: Frames in a heated debate. Science Communication 24(2): 184–197.

[bibr10-0963662520907255] Davis PR Russ RS (2015) Dynamic framing in the communication of scientific research: Texts and interactions. Journal of Research in Science Teaching 52(2): 221–252.

[bibr11-0963662520907255] de Vreese CH (2005) News framing: Theory and typology. Information Design Journal & Document Design 13(1): 51–62.

[bibr12-0963662520907255] de Saille S (2015) Innovating innovation policy: The emergence of ‘Responsible Research and Innovation’. Journal of Responsible Innovation 2(2): 152–168.

[bibr13-0963662520907255] Delfanti A (2013) Biohackers: The Politics of Open Science. London: Pluto Press.

[bibr14-0963662520907255] Delvenne P (2017) Responsible research and innovation as a travesty of technology assessment? Journal of Responsible Innovation 4(2): 278–288.

[bibr15-0963662520907255] Endy D (2005) Foundations for engineering biology. Nature 438(7067): 449–453. 16306983 10.1038/nature04342

[bibr16-0963662520907255] Frow E (2017) From ‘experiments of concern’ to ‘groups of concern’: Constructing and containing citizens in synthetic biology. Science, Technology, & Human Values. Epub ahead of print 25 October 2017. DOI: 10.1177/0162243917735382.

[bibr17-0963662520907255] Gamson WA Modigliani A (1989) Media discourse and public opinion on nuclear power: A constructionist approach. American Journal of Sociology 95(1): 1–37.

[bibr18-0963662520907255] Habermas J (1970) Toward a Rational Society: Student Protest, Science, and Politics. Boston, MA: Beacon Press.

[bibr19-0963662520907255] Hajer M Laws D (2006) Ordering through discourse. In: Goodin RE Moran M Rein M (eds) The Oxford Handbook of Public Policy. London: Oxford University Press, pp. 251–268.

[bibr20-0963662520907255] Jasanoff S (2003) (No?) Accounting for expertise. Science and Public Policy 30(3): 157–162.

[bibr21-0963662520907255] Jasanoff S Kim S-H (eds) (2015) Dreamscapes of Modernity: Sociotechnical Imagineries and the Fabrication of Power. Chicago, IL: Unversity of Chicago Press.

[bibr22-0963662520907255] Levidow L Carr S (2007) GM crops on trial: Technological development as a real-world experiment. Futures 39(4): 408–431.

[bibr23-0963662520907255] Macnaghten P Davies SR Kearnes M (2015) Understanding public responses to emerging technologies: A narrative approach. Journal of Environmental Policy & Planning 21(5): 504–518.

[bibr24-0963662520907255] Maeseele PA Schuurman D (2008) Biotechnology and the popular press in northern Belgium: A case study of hegemonic media discourses and the interpretive struggle. Science Communication 29(4): 435–471.

[bibr25-0963662520907255] Navid EL Einsiedel EF (2012) Synthetic biology in the Science Café: What have we learned about public engagement? Journal of Science Communication 11(4): A02.

[bibr26-0963662520907255] Nisbet MC (2009) Communicating climate change: Why frames matter for public engagement. Environment 51(2): 12–23.

[bibr27-0963662520907255] Nisbet MC (2010) Framing science: A new paradigm in public engagement. In: Kahlor L Stout P (eds) Communicating Science. New Agendas in Communication. New York; London: Routledge, pp. 40–67.

[bibr28-0963662520907255] Nisbet MC Lewenstein BV (2002) Biotechnology and the American media: The policy process and the elite press, 1970 to 1999. Science Communication 23(4): 359–391.

[bibr29-0963662520907255] Nisbet MC Brossard D Kroepsch A (2003) Framing science: The stem cell controversy in an age of press/politics. Harvard International Journal of Press/Politics 8(2): 36–70.

[bibr30-0963662520907255] Owen R Macnaghten P Stilgoe J (2012) Responsible research and innovation: From science in society to science for society, with society. Science and Public Policy 39(6): 751–760.

[bibr31-0963662520907255] Pan Z Kosicki GM (1993) Framing analysis: An approach to news discourse. Political Communication 10(1): 55–75.

[bibr32-0963662520907255] Pinch TJ Bijker WE (1984) The social construction of facts and artefacts: Or how the sociology of science and the sociology of technology might benefit each other. Social Studies of Science 14(3): 399–441.

[bibr33-0963662520907255] Scheufele DA Tewksbury D (2006) Framing, agenda setting, and priming: The evolution of three media effects models. Journal of Communication 57(1): 9–20.

[bibr34-0963662520907255] Schmidt M Meyer A Cserer A (2015) The BioFiction film festival: Sensing how a debate about synthetic biology might evolve. Public Understanding of Science 24(5): 619–635. 24164747 10.1177/0963662513503772 PMC4466099

[bibr35-0963662520907255] Scholte S Vasileiadou E Petersen AC (2013) Opening up the societal debate on climate engineering: How newspaper frames are changing. Journal of Integrative Environmental Sciences 10(1): 1–16.

[bibr36-0963662520907255] Schön DA Rein M (1994) Frame Reflection: Toward the Resolution of Intractable Policy Controversies. New York: Basic Books.

[bibr37-0963662520907255] Swierstra T Rip A (2007) Nano-ethics as NEST-ethics: Patterns of moral argumentation about new and emerging science and technology. Nanoethics 1(1): 3–20.

[bibr38-0963662520907255] Torgersen H Schmidt M (2013) Frames and comparators: How might a debate on synthetic biology evolve? Futures 48: 44–54. 23805003 10.1016/j.futures.2013.02.002 PMC3688360

[bibr39-0963662520907255] van Gorp B (2007) The constructionist approach to framing: Bringing culture back. Journal of Communication 57(1): 60–78.

[bibr40-0963662520907255] van Gorp B (2010) Strategies to take subjectivity out of framing analysis. In: D’Angelo P Kuypers JA (eds) Doing News Framing Analysis: Empirical and Theoretical Perspectives. New York; London: Routledge, pp. 100–125.

[bibr41-0963662520907255] van Hulst M Yanow D (2014) From policy ‘frames’ to ‘framing’: Theorizing a more dynamic, political approach. The American Review of Public Administration 46(1): 92–112.

[bibr42-0963662520907255] von Schomberg R (2013) A vision of responsible research and innovation. In: Owen R Bessant J Heintz M (eds) Responsible Innovation: Managing the Responsible Emergence of Science and Innovation in Society. Chichester: John Wiley & Sons, pp. 51–74.

[bibr43-0963662520907255] Wynne B (2007) Public participation in science and technology: Performing and obscuring a political-conceptual category mistake. East Asian Science, Technology and Society 1(1): 99–110.

