# Supplementary material for: Let’s (not) talk about synthetic biology: Framing an emerging technology in public and stakeholder dialogues
Source: Public Underst Sci. 2020 Mar 3;29(5):492–507. doi: 10.1177/0963662520907255 (PMC7411530; doi:10.1177/0963662520907255)
Supplement: framing_synbio_observation_protocol – Supplemental material for Let’s (not) talk about synthetic biology: Framing an emerging technology in public and stakeholder dialogues [file framing_synbio_observation_protocol.pdf]

Let’s (not) talk about synthetic biology: Framing an emerging technology in public and stakeholder dialogues – Supplemental Material

Anja Bauer[a,b], Alexander Bogner[b]

[a] Department of Science, Technology and Society Studies, Alpen-Adria-Universität Klagenfurt, Universitätsstraße 65-67, 9020 Klagenfurt, Austria, anja.bauer@aau.at

[b] Institute of Technology Assessment, Austrian Academy of Sciences, Apostelgasse 23, 1030 Vienna, Austria, anja.bauer@oeaw.ac.at, abogner@oeaw.ac.at

Synenergene - Observation Protocol

[Generic, may be adapted to the specifics of the event]

A Background information

Name of the event	
Date of the event	
Venue of the event	
Context of the event (i.e. is it part of a series of events, did participants meet previously?)	
Organisers of the event	
Format(s) of the event (e.g. science café, workshop, theatre performance)	
Stated aim(s) of the event (by organisers)	

B Observation of setting and interactions

**1. Setting & procedure**	
Physical setting of the event, i.e. how the room and seating of participants are arranged (e.g. separated podium and audience; several tables for small group discussions, visualization tools, etc.).	
Procedure of the event, i.e. how does the event start (introduction by the organiser, experts' input, etc.), what are the single phases of the event?	

**2. Actors and roles**	
Approx. <u>number of participants</u>	
Distribution along age and gender	
How were the participants <u>invited</u>? (By whom, by which means?)	
Which <u>roles</u> are assigned to participants (or taken by themselves)? Are these roles kept strictly or do they change or dissolve during the event?<br>What is the <u>role of the moderator?</u> In how far does the moderator guide the debate?	
**3. Forms and intensity of interaction**	
Overall: How much room is provided for presentations and questions from the audience?	
In how far does a <u>genuine dialogue</u> emerge? (Do participants react on the statements of others, do they really relate to each other? Or do participants provide isolated statements that only loosely relate to the statements of other participants?)	
How do participants <u>present their positions</u> in the debate? With emphasis and passion or in a more distant way taking different aspects into account ("on the one hand … on the other…")?	
Are there <u>participants who dominate</u> the discussions (i.e. make considerable more statements than others, have considerably more speaking time or are able to guide the further discussion by their arguments more than others)? Are there participants who hardly contribute?	
What is the overall <u>character of the debate</u> (e.g. consensual versus adversarial; abstract/generic versus concrete, rational/detached versus emotional?)<br>Does the character of the debate change over the course of the event?	

B Contents of the event (issue & frames)

1. **Transcript**

Transcribe the event in its sequence (separate sheets), note who said what and how!<br><br>*Please take especially the following aspects into account:*<br><br>*How is synthetic biology or the more specific issue (e.g. gene drives) introduced and presented and by whom?*<br><br>*What are the main issues and aspects that are discussed during the event?*<br><br>*With regard to the latter, pay particular attention to catch words and metaphors such as:*<br><br>*Playing god, human dignity, autonomy, justice, harm, danger, environment, grand challenges, sustainable, open access, welfare, democratisation, expertise, information, objective, neutral.*

2. **Summary and reflection (immediately written after the event)**

In how far were the issues discussed pre-defined through the agenda? In how far did participants introduce new topics and aspects on their own?	
What issues were widely agreed on by participants? Which issues were contentious among participants? (if possible state the consensus or different positions)	
